# Variability in the common genetic architecture of social-communication spectrum phenotypes during childhood and adolescence

**DOI:** 10.1186/2040-2392-5-18

**Published:** 2014-02-24

**Authors:** Beate St Pourcain, David H Skuse, William P Mandy, Kai Wang, Hakon Hakonarson, Nicholas J Timpson, David M Evans, John P Kemp, Susan M Ring, Wendy L McArdle, Jean Golding, George Davey Smith

**Affiliations:** 1 The Medical Research Council Integrative Epidemiology Unit, University of Bristol, Oakfield House, Oakfield Grove, Bristol BS8 2BN, UK; 2 School of Oral and Dental Sciences, University of Bristol, Lower Maudlin Street, Bristol BS1 2LY, UK; 3 School of Experimental Psychology, University of Bristol, 12a Priory Road, Bristol BS8 1TU, UK; 4 Behavioural Sciences Unit, Institute of Child Health, University College London, Gower Street, London WC1E 6BT, UK; 5 Research Department of Clinical, Educational and Health Psychology, University College London, Gower Street, London WC1E 6BT, UK; 6 Children’s Hospital of Philadelphia and Perelman School of Medicine, 3615 Civic Center Boulevard, Philadelphia, PA 19104, USA; 7 Zilkha Neurogenetic Institute & Department of Psychiatry, Keck School of Medicine of the University of Southern California, 1501 San Pablo St, Los Angeles, CA 90089, USA; 8 University of Queensland Diamantina Institute, Level 7, 37 Kent St, Translational Research Institute, Woolloongabba QLD 4102, Australia; 9 School of Social and Community Medicine, University of Bristol, Oakfield House, Oakfield Grove, Bristol BS8 2BN, UK; 10 Centre for Child and Adolescent Health, University of Bristol, Oakfield House, Oakfield Grove, Bristol BS8 2BN, UK

**Keywords:** ALSPAC, ASD, Autism, GCTA heritability, GWAS, Social communication

## Abstract

**Background:**

Social-communication abilities are heritable traits, and their impairments overlap with the autism continuum. To characterise the genetic architecture of social-communication difficulties developmentally and identify genetic links with the autistic dimension, we conducted a genome-wide screen of social-communication problems at multiple time-points during childhood and adolescence.

**Methods:**

Social-communication difficulties were ascertained at ages 8, 11, 14 and 17 years in a UK population-based birth cohort (Avon Longitudinal Study of Parents and Children; *N* ≤ 5,628) using mother-reported Social Communication Disorder Checklist scores. Genome-wide Complex Trait Analysis (GCTA) was conducted for all phenotypes. The time-points with the highest GCTA heritability were subsequently analysed for single SNP association genome-wide. Type I error in the presence of measurement relatedness and the likelihood of observing SNP signals near known autism susceptibility loci (co-location) were assessed via large-scale, genome-wide permutations. Association signals (*P* ≤ 10^−5^) were also followed up in Autism Genetic Resource Exchange pedigrees (*N* = 793) and the Autism Case Control cohort (*N*_cases_/*N*_controls_ = 1,204/6,491).

**Results:**

GCTA heritability was strongest in childhood (*h*^2^_(8 years)_ = 0.24) and especially in later adolescence (*h*^2^_(17 years)_ = 0.45), with a marked drop during early to middle adolescence (*h*^2^_(11 years)_ = 0.16 and h^2^_(14 years)_ = 0.08). Genome-wide screens at ages 8 and 17 years identified for the latter time-point evidence for association at 3p22.2 near *SCN11A* (rs4453791, *P* = 9.3 × 10^−9^; genome-wide empirical *P* = 0.011) and suggestive evidence at 20p12.3 at *PLCB1* (rs3761168, *P* = 7.9 × 10^−8^; genome-wide empirical *P* = 0.085). None of these signals contributed to risk for autism. However, the co-location of population-based signals and autism susceptibility loci harbouring rare mutations, such as *PLCB1*, is unlikely to be due to chance (genome-wide empirical *P*_co-location_ = 0.007).

**Conclusions:**

Our findings suggest that measurable common genetic effects for social-communication difficulties vary developmentally and that these changes may affect detectable overlaps with the autism spectrum.

## Background

Psychological theories understand autism as a dimensional disorder, with autism spectrum disorders (ASDs) delineating the extreme end of a continuum reflecting developmental difficulties [1,2]. Symptoms include deficits in social interaction and communication as well as highly restricted interests and/or stereotyped, repetitive behaviours [3]. Support for the existence of dimensionally varying ASD related phenotypes has been provided both through the identification of subclinical traits in family members of autistic patients [4] and by studies demonstrating the continuous distribution of autistic symptoms in the general population [5,6]. Twin studies have reported that these quantitative non-psychopathological traits, including social-communication difficulties, show evidence of heritability (*h*^2^ = 0.36 to 0.87) [7]. However, little is known about the underlying molecular nature of these ASD related phenotypes in the general population or their genetic links with the autistic dimension.

Currently, there is no twin research-based evidence for differences in heritability estimates of autistic symptomatology at either end of the autism continuum [8,9], suggesting that clinical ASD and autistic traits may have a common aetiology. This notion has been supported by genetic association studies, which have indeed identified links between autistic traits, including social-communication spectrum phenotypes, and selected ASD risk loci, such as common variation at 5p14 [10], *CNTNAP2*[11], *CYP11B1*[12] and *NTRK1*[12]. However, it is not known how representative these findings are within a genome-wide context, especially as there is currently little evidence for large effects of individual common variants on the risk for ASD [13]. In particular, recently reported genome-wide association studies (GWASs) for both ASD [13-15] and autistic traits [16,17], including social-communication problems [17], failed to show signals which could either be replicated [18] or that could be considered genome-wide significant. It is therefore possible that the observed common single-nucleotide polymorphism (SNP) associations linking autistic traits and ASD are the exception rather than the rule.

Recent work has shown however the importance of rare structural variation within the genetic architecture of ASD. Rare *de novo* mutations have been repeatedly observed in 5% to 10% of all affected individuals [19,20], and the most pronounced risk for ASD has been attributed to large, multigenic, *de novo* copy number variations, which is many times greater than the risk assumed for common variants [21].

In light of these findings, it might be unsound to assume that the genetic mechanisms affecting, for example, subtle impairments in social-communication skills and the risk of a severe childhood developmental disorder are shared for the majority of loci involved. However, this does not preclude the existence of overarching functional mechanisms connecting the extreme and subclinical ends of the continuum. With regard to many traits, it has been shown that in genes which, when mutated, cause severe phenotypic perturbation, common variants exist that have smaller effects on the same phenotype [22,23]. As such, both ends of the autistic dimension may indeed share functionality involving the same gene locus, although the genetic mechanisms implicating the locus in the phenotype expression may fundamentally vary. However, little is known to date about the co-location of common social-communication related signals within the vicinity of ASD loci carrying, for example, rare mutations.

Under the assumption that links between common social-communication related genetic variation and ASD loci indeed exist, the power to detect such an overlap will depend on the selection of the genetically most stable and enriched population-based phenotype. For instance, an increase in heritability from childhood to young adulthood, such as was recently demonstrated for general cognitive ability [24], would imply that genes might be easier to study in adults than in children. Both, diagnoses of ASD and high scores on autistic trait measures, have been shown to be highly persistent throughout development [25], and the results of twin studies have suggested a similar aetiology for the general population and the extreme end of the autism continuum [8,9]. Therefore, it could also be assumed that the contribution of genetic variants to phenotypic variance in social-communication traits remains stable during development. To date, however, this has never been demonstrated developmentally, especially for quantitative traits. Moreover, the analysis of a related continuous phenotype, language skills, identified age related changes in heritability during mid-adolescence [26], especially for indirectly assessed measures, suggesting that also the heritability of social-communication traits may be subject to temporal variation.

Adopting a developmental perspective, we conducted a GWAS of impaired social-communication traits within the general population at multiple time-points during childhood and adolescence with the aim of assessing both common joint additive and common single SNP effects genome-wide. We analysed a large UK population-based birth cohort, the Avon Longitudinal Study of Parents and Children (ALSPAC), for which the continuity of ASD related traits has been demonstrated [27]. The strongest SNP signals were eventually studied in autism samples, and the likelihood of co-location with a potential ASD locus was assessed through permutations.

## Methods

### Study population for genome-wide analysis

Genome-wide analysis was performed using participants from ALSPAC, a UK population-based, longitudinal, pregnancy-ascertained birth cohort (estimated birth dates between April 1991 and December 1992) [28]. The cohort is representative of the general population (approximately 96% White mothers). Ethical approval was obtained from the ALSPAC Law-and-Ethics Committee (IRB00003312) and the Local Research Ethics Committees, and written informed consent was provided by all parents. The study website contains details of all available data (http://www.bris.ac.uk/alspac/researchers/data-access/data-dictionary).

### Measurement of social-communication problems

Social-communication problems in ALSPAC children were captured with the 12-item Social Communication Disorder Checklist (SCDC) [29], which has a score range from 0 to 24. The SCDC is a brief screening instrument of social reciprocity, and verbal and nonverbal communication [29] (age range from 3 to 18 years) that has high sensitivity and specificity for autism, with higher scores reflecting more social-communication deficits. Thus, the instrument was employed to capture the dimensionality of social-communication traits. Mother-reported SCDC scores for children and adolescents were measured at 8, 11, 14 and 17 years of age and showed high temporal stability (0.38 < ρ < 0.58) (Table 1 and Additional file 1: Table S1).

**Table 1:** Phenotype description and Genome-wide Complex Trait Analysis

	**Age (years)**			
**Variables**	**8**	**11**	**14**	**17**
SCDC score description				
Median (SD) [range]	2 (3.71) [0 to 24]	1 (3.51) [0 to 24]	1 (3.60) [0 to 24]	1 (3.79) [0 to 24]
Age, years (SD)	7.65 (0.14)	10.72 (0.13)	13.90 (0.15)	16.84 (0.36)
Males (%)	51.2%	50.3%	50.0%	48.4%
*N*_*Total*_^a^	5,628	5,533	5,129	4,229
SCDC GCTA heritability^b^				
*h*^2^ (SE)	0.24 (0.07)	0.16 (0.07)	0.08 (0.07)	0.45 (0.08)
LRT (*df*)	14.26 (1)	6.33 (1)	1.38 (1)	33.69 (1)
*P*-value	8.0 × 10^−5^	5.9 × 10^−3^	0.12	3.2 × 10^−9^
*N*_*GCTA*_^c^	5,204	5,121	4,797	4,026

Phenotype description and GCTA within ALSPAC; GCTA, Genome-wide Complex Trait Analysis; LRT, Likelihood ratio test; SCDC, Social Communication Disorder Checklist; ALSPAC, Avon Longitudinal Study of Parents and Children; ^a^Individuals with genotypic and phenotypic data. ^b^GCTA heritability estimates based on rank-transformed SCDC scores (adjusted for age and sex and the two most significant ancestry-informative principal components). ^c^Individuals subjected to GCTA, differences compared with the total sample (*N*_*Total*_) are due to the exclusion of individuals with relatedness ≥2.5% in GCTA.

### Genotyping and imputation

ALSPAC participants (*N* = 9,912) were genotyped using the Illumina HumanHap550-Quad BeadChip genotyping platform (Illumina, Cambridge, UK; 609,203 probes, including approximately 60,000 custom probes) by 23andMe (Mountain View, CA, USA) at either the Wellcome Trust Sanger Institute (Cambridge, UK) or the Laboratory Corporation of America (Burlington, NC, USA). Data were cleaned using standard quality control methods as previously described [30]. In brief, SNPs with a minor allele frequency (MAF) <1%, a call rate <95% or evidence of violations of Hardy–Weinberg equilibrium (*P* < 5.0 × 10^−7^) were removed. Individual samples were excluded on the basis of sex mismatches, minimal or excessive heterozygosity, disproportionate levels of individual missingness (>3%), cryptic relatedness (>10% identity by descent), insufficient sample replication and non-European ancestry. This resulted in 464,311 directly genotyped SNPs for 8,365 independent individuals (irrespective of available phenotypic data), which were imputed to HapMapCEU (Utah Residents with Northern and Western European Ancestry from the Centre d’Etude du Polymorphisme Humain collection) (release 22) using MaCH [31]. Subtle differences in population structure were adjusted for by using principal components calculated with EIGENSTRAT [32]. All reported linkage disequilibrium (LD) measures were based on HapMapCEU (release 22).

### Estimation of heritability, genetic and environmental-residual correlations using Genome-wide Complex Trait Analysis

Using Genome-wide Complex Trait Analysis (GCTA) [33], the proportion of additive phenotypic variation explained by all SNPs together (GCTA heritability) was estimated for SCDC scores at 8, 11, 14 and 17 years. Pertinent to this study, we used rank-transformed (and thus normally distributed) residuals of social-communication traits adjusted for age, sex and the first two ancestry-informative principal components, and 464,311 directly genotyped SNPs. Note that all analyses were adjusted for age, as we observed an association between age and SCDC scores at age 14 years (*P* = 5.1 × 10^−6^), although there was no such evidence at any other time-point (data not shown). Bivariate GCTA [34] was performed to estimate genetic correlations and environmental-residual correlations between time-points. Phenotypic correlations between transformed data were based on Pearson product–moment coefficients (Additional file 1: Table S1 and Additional Note for GCTA details).

### Genetic association analysis

Single time-point genome-wide screens were conducted on approximately 2.3 million (*N* = 2,293,137) imputed and genotyped SNPs with high imputation accuracy (MaCH *R*^2^ > 0.8) using the phenotypes with the highest GCTA heritability, i.e. SCDC scores at ages 8 and 17 years (see below). Association analyses were performed using quasi-Poisson regression, which can accommodate overdispersion [35] (R software package ‘stats’ and ‘speedglm’ libraries). Specifically, untransformed (and thus directly interpretable) counts of social-communication problems were regressed on age, sex, the two most significant ancestry-informative principal components and allele dosage. Regression estimates (β) represent changes in log counts per effect allele. All single time-point findings were subjected to genomic-control (GC) correction.

In order to account (I) for type I error and the non-independence of the performed single time-point genome-wide screens, and to assess (II) the likelihood of observing common social-communication related signals within the vicinity of autism susceptibility loci, we carried out permutations (*S* = 1,000) on a high-performance computing machine, BlueCrystal Phase 2 (https://www.acrc.bris.ac.uk/phase2.htm). Similar large-scale permutation approaches to assess observed genome-wide association signals have been successfully applied before [36]. Within our study, we reduced the total number of analysed SNPs (*N* = 2,293,137) to a set of independent index variants (*N* = 201,028) based on LD (±500 kb, *r*^2^ = 0.3, using the PLINK whole genome analysis toolset [37], pruning the GWAS with the most strongly associated single signals, that is, the time-point at age 17 years). This step eased the computational burden while permutations remained exchangeable under the null hypothesis. Furthermore, the aggressive LD pruning controlled for the possibility of any bias due to LD, which is inherent to some permutation-based approaches [38]. For the permutation analysis, all phenotypic data were jointly permuted together as a vector, including the SCDC scores at ages 8 and 17 years. Empirical genome-wide significance accounting for non-independence (I) was based on the number of times any signal from any permuted single time-point GWAS (that is, at ages 8 and 17 years) would pass the selected threshold. This threshold was based on the GC corrected *P*-value for the most significantly associated SNP signals at age 17 years, that is, *P* = 9.3 × 10^−9^ and *P* = 7.9 × 10^−8^ for the 3p22.2 and 20p12.3 signal, respectively.

Furthermore, each clumped LD region (associated with one of the 201,028 index SNPs) was aligned to a set of autism candidate loci (375 autosomal loci; SFARI Gene https://gene.sfari.org/autdb/Welcome.do) representing approximately 2% of all known genes (hg18). For the co-location analysis (II) (that is, assessing the probability that a GWAS signal is randomly observed within the vicinity of a known ASD locus), we additionally determined how many times the clumped LD region for signals passing the selection threshold also harboured at least one autism susceptibility gene. Permutation-based standard errors (SEs) were based on the Binomial distribution.

For sensitivity analysis, the strongest single time-point GWAS signals were characterised longitudinally using mixed Poisson regression (R statistical software package ‘lme4’ library), where overdispersion can be modelled through the random error part [39]. Models were fitted using full maximum likelihood with age as a continuous variable incorporating all available data at each time-point. We included random effects for intercept and slope (age), capturing measurement variation within individuals, as well as fixed effects for SNP allele dosage, age, sex and age × sex interactions, and, if required, age × SNP interactions. The most parsimonious model fit was assessed through likelihood ratio tests.

### Gene expression analysis

RNA expression was studied in up to 875 unrelated ALSPAC individuals for which lymphoblastoid cell lines (LCLs) had been generated. LCLs were grown until confluent, and cells were frozen in RNAlater reagent (QIAGEN, Manchester, UK). RNA from LCLs was extracted using the RNeasy extraction kit (QIAGEN) and amplified using the TotalPrep-96 RNA Amplification Kit (Illumina). Expression was surveyed using the HumanHT-12 v3 Expression BeadChip array (Illumina). Each sample was run with two replicates. Expression data were normalised using quantile normalisation between replicates and then median normalisation across all analysed individuals. For the statistical analyses, all measurements were rank-transformed. Linear regression was used to investigate the relationship between SNP variation and changes in cis transcript expression of nearby loci.

### Autism spectrum disorder sample and association analysis

Population-based signals were followed up for association with ASD in the Autism Genetic Resource Exchange (AGRE) pedigrees and the Autism Case-Control (ACC) cohort. Ethical approval for the analysis of the AGRE and ACC samples was obtained through the IRB Protocol 10–007590 from the Children's Hospital of Philadelphia. The analysis involved only de-identified genetic data. Within the multiethnic AGRE, there are three diagnostic categories based on the Autism Diagnostic Interview–Revised (ADI–R) [40]: Autism, Broad Spectrum or Not Quite Autism, which have been described previously in detail [14]. In total, 4,444 unique individuals from 943 families were genotyped on the Illumina HumanHap550K BeadChip containing over 550,000 SNPs [14]. Cleaned genome-wide association data [14] were obtained from Autism Speaks (data set prepared by JK Lowe). In brief, this data cleaning involved the removal of SNPs with >10% missingness, violations of Hardy–Weinberg equilibrium (*P* < 0.001), MAF <1% and more than 10 Mendelian errors, as well as the exclusion of monozygotic twins, sample duplicates and individuals with >10% missing data. After these data were excluded, 4,327 individuals and 513,312 SNPs remained in our data set. We also removed individuals with known chromosomal abnormalities (including Trisomy 21 and Fragile X syndrome). In addition, we restricted the analysis to individuals of European ancestry using multidimensional scaling (MDS) as implemented in PLINK [37]. Using the first two principal components identified by MDS, we included all individuals with values between −0.021 and 0.005 for the first component and values between −0.020 and 0.020 for the second component. This resulted in a final data set of 3,299 individuals (793 pedigrees) and 513,312 SNPs. Genotypes were imputed to HapMapCEU (release 22) using MaCH, excluding all imputed calls with a per-genotype posterior probability <0.9. For the follow-up of selected SNPs, an association analysis was performed with FBAT, a family-based association test [41], using the most likely genotypes. An empirical variance for the test statistic was selected to account for linkage within pedigrees.

The ACC cohort comprised 1,453 affected individuals with either a positive ADI/ADI–R score or an Autism Diagnostic Observation Schedule [42] diagnosis or both, in addition to 7,070 control children without a history of ASD. Genome-wide data (Illumina HumanHap550K BeadChip with over 550,000 SNPs) were obtained for all individuals as previously described [14], and the data cleaning was largely similar to the cleaning of the AGRE sample [14] (see above). After quality control, the final data set comprised 1,204 ASD cases and 6,491 controls of European ancestry, as well as 480,530 SNPs [14]. Genotypic data were imputed to HapMapCEU (release 22) using MaCH as previously reported [14]. An association analysis of selected follow-up SNPs was carried out using SNPTEST [43] by converting MaCH imputation files into SNPTEST input formats [14].

## Results

### Genome-wide analysis

**Figure 1:**
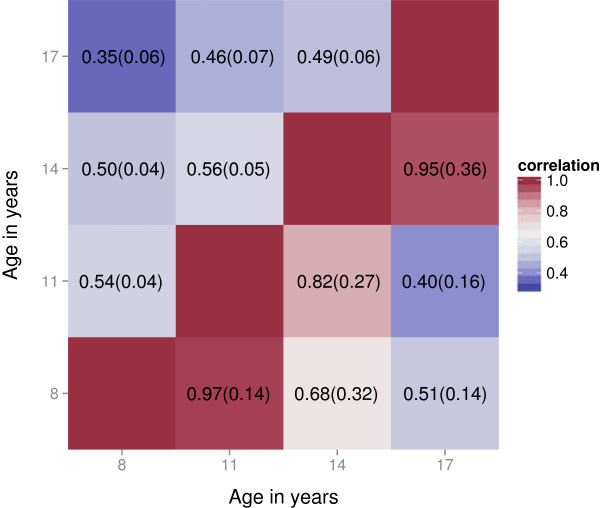
**Genetic and environmental-residual correlations between social-communication problems during development.** Lower triangle: genetic correlations (*r*_g_). Upper triangle: environmental-residual correlations (*r*_e_). All correlations are given with their SE.

Social-communication scores at the ages of 8, 11, 14 and 17 years were highly interrelated, with most ALSPAC children and adolescents showing few problems during the course of development (Table 1; Additional file 1: Table S1). Using rank-transformed SCDC scores, GCTA heritability estimates (Table 1) were strongest during childhood (*h*^2^_(8 years)_ = 0.24 (SE = 0.07); *P* = 8.0 × 10^−5^) and especially during later adolescence (*h*^2^_(17 years)_ = 0.45 (SE = 0.08); *P* = 3.2 × 10^−9^), whereas there was little or no evidence for joint additive genetic effects during early to middle adolescence (*h*^2^_(11 years)_ = 0.16 (SE = 0.07), *P* = 5.9 × 10^−3^; *h*^2^_(14 years)_ = 0.08 (SE = 0.07), *P* = 0.12). Genetic correlations based on GCTA (0.40 < *r*_g_ ≤ 0.97, 2 × 10^−7^ < *P* ≤ 0.04; Figure 1 and Additional file 1: Table S2), however, showed that common genetic variation is shared developmentally, especially between adjacent time-points (0.82 < *r*_g_ ≤ 0.97) but also at more distant time-points (*r*_g(8–17 years)_ = 0.51). In comparison, environmental-residual correlations were considerably lower (0.35 < *r*_e_ ≤ 0.56, Figure 1).

**Figure 2:**
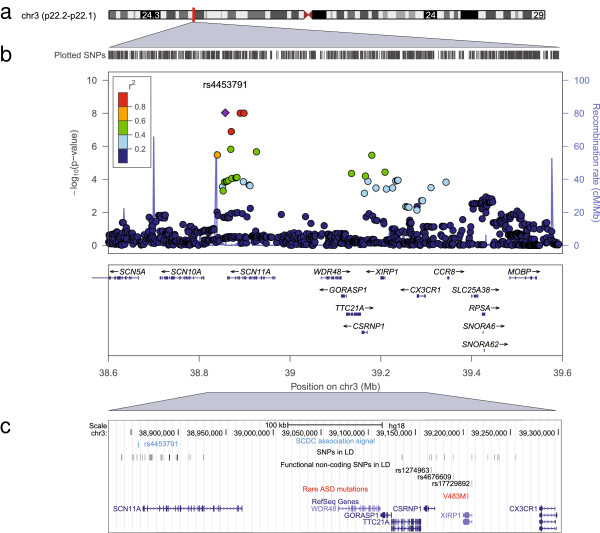
**Association signal at 3p22.2 for social-communication problems at 17 years of age. (a)** Chromosome ideogram for chromosome 3. **(b)** Regional association plot for rs4453791. Directly genotyped and imputed variants are depicted by filled circles according to their genome-wide -log_10_*P*-value and their genomic position in megabases (Mb) (build 36). Recombination rates are shown in blue (HapMapCEU, release 22) and the linkage disequilibrium (LD) (*r*^2^) between the lead variant and surrounding markers is indicated by the colour code. **(c)** Detailed genomic region near rs4453791 depicting variants in LD (*r*^2^ > 0.3), non-coding functional variation (ENCODE (Encyclopedia of DNA Elements) functionality score ≤2; RegulomeDB: http://regulome.stanford.edu/) and a rare autism related *de novo* single-nucleotide variant (V483M) within *XIRP1*, a locus with weak candidacy [44]. The LD (*r*^2^) between the lead variant and surrounding SNPs is indicated by the shade of grey (0 (white) to 1 (black)).

**Figure 3:**
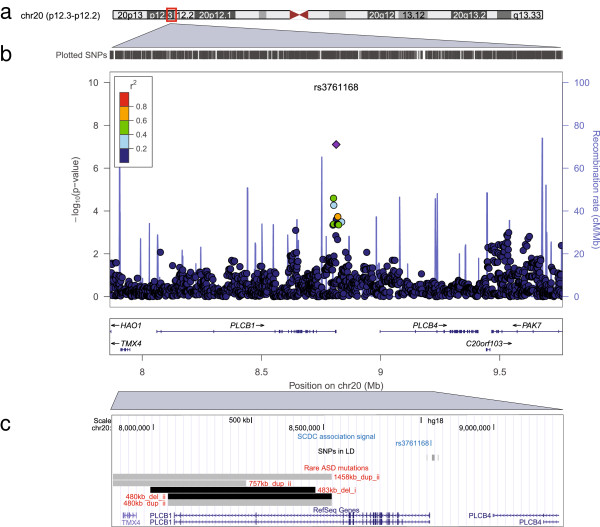
**Association signal at 20p12.3 for social-communication problems at 17 years of age. (a)** Chromosome ideogram for chromosome 20. **(b)** Regional association plot for rs3761168. Directly genotyped and imputed variants are depicted by filled circles according to their genome-wide -log_10_*P*-value and their genomic position in megabases (Mb) (build 36). Recombination rates are shown in blue (HapMapCEU, release 22), and the linkage disequilibrium (LD) (*r*^2^) between the lead variant and surrounding markers is indicated by the colour code. **(c)** Detailed genomic region near rs3761168 depicting variants in LD (*r*^2^ > 0.3) and rare autism related mutations (≥480 kb) within *PLCB1*. Mutations were either (i) identified in affected family members only [45] or (ii) enriched within patients compared with controls [46] (del, deletions; dup, duplications). The LD (*r*^2^) between the lead variant and surrounding SNPs is indicated by the shade of grey (0 (white) to 1 (black)).

To enhance the power of the genome-wide screen, our GWAS was carried out using the phenotypes with the highest GCTA heritability, i.e. SCDC scores at ages 8 and 17 years (Additional file 1: Table S3). This revealed an excess of association signals beyond chance at age 17 years only and little evidence for population stratification at either time-point (1.03 < λ_GC_ ≤ 1.04; Additional file 1: Figure S1). The strongest association signal was observed at rs4453791 residing approximately 5 kb near the 3′ end of the voltage-gated type XI sodium-channel α gene (*SCN11A*) on chromosome 3p22.2 (GC corrected *P* = 9.3 × 10^−9^, genome-wide empirical *P* = 0.011 (SE = 0.0033); Table 2, Figure 2 and Additional file 1: Table S3). Specifically, each increase in C allele at rs4453791 was associated with an increase in 0.23 log counts of social-communication difficulties. The second strongest signal, approaching near genome-wide significance (GC-corrected *P* = 7.9 × 10^−8^; genome-wide empirical *P* = 0.085 (SE = 0.0088); Table 2, Figure 3 and Additional file 1: Table S3), was identified at rs3761168 on chromosome 20p12.3. This SNP locates 170 bp near the 5′ end of the phospholipase C (β1) (*PLCB1*) gene and was associated with an increase of 0.32 log counts in social-communication problems per A allele.

**Table 2:** Strongest association signals for social-communication problems across developmental stages

					**8 years (*N*** **= 5,628)**		**11 years (*N*** **= 5,533)**		**14 years (*N*** **= 5,129)**		**17 years (*N*** **= 4,229)**		**Empirical GWAS *P*-value (SE)**	**Empirical GWAS *P*-value (SE) for co-location with autism loci**
**SNP**	**Chr**	**Gene**	**E,A**	**EAF**	**β (SE)**^**a**^	***P***^**a**^	**β (SE)**	***P***	**β (SE)**	**P**	**β (SE)**^**a**^	***P***^**a**^		
rs4453791	3	*SCN11A*	c,t	0.13	0.03 (0.04)	0.48	0.05 (0.04)	0.27	0.13 (0.04)	0.0014	**0.23 (0.04)**	**9.3 × 10**^**−9**^	**0.011 (0.0033)**	**0.002 (0.0014)**
rs3761168	20	*PLCB1*	a,c	0.05	0.12 (0.05)	0.025	0.17 (0.06)	0.0041	0.09 (0.06)	0.16	0.32 (0.06)	7.9 × 10^−8^	0.085 (0.0088)	**0.007 (0.0026)**

GWAS results for SCDC scores at 8 and 17 years of age are presented for the most significant independent signals (Genomic-control corrected single time-point *P* < 1 × 10^−7^). Regression estimates were obtained using quasi-Poisson regression analysis. SNPs were imputed with sufficient quality (rs4453791: MaCH *R*^2^ = 0.98, rs3761168: MaCH *R*^2^ = 0.96). Genome-wide significant results are indicated in bold. In addition, association results at the selected signals are also presented for SCDC scores at 11 and 14 years of age (not subjected to GWAS). A, Alternative allele; Chr, Chromosome; E, Effect allele; EAF, Effect allele frequency; Empirical GWAS-P (SE), Empirical genome-wide *P*-value based on 1,000 permutations accounting for multiple testing at ages 8 and 17 years; Empirical GWAS-P (SE) for co-location with autism loci, Empirical genome-wide *P*-value based on 1,000 permutations accounting for multiple testing at ages 8 and 17 years and conditional on the entire set of currently known putative autism loci (LD-based gene region: *r*^2^ > 0.3, ±500 kb, 375 autosomal genes; https://gene.sfari.org/autdb/Welcome.do). Gene, Nearest gene within ±500 kb of the SNP; GWAS, Genome-wide Association Study; SNP, Single-nucleotide polymorphism. ^a^Genomic-control corrected during GWAS.

### Longitudinal assessment of genetic effects

Single time-point analysis showed a continuous increase in genetic influences at rs4453791 during development, rising from 0.03 log counts per risk allele at age 8 years to 0.23 log counts at age 17 years (Table 2). This was reflected by an age × SNP interaction effect (β = 0.018 (SE = 0.0054); *P* = 0.0013) when data were modelled longitudinally (Additional file 1: Table S4). The genetic effect at rs3761168 was less consistent across development (Table 2). Longitudinal analyses (Additional file 1: Table S4) suggested the presence of a time-independent effect (β = 0.17 (SE = 0.053), *P* = 9.8 × 10^−4^), with no evidence for an age × SNP interaction (*P* = 0.22, data not shown). We found no support for SNP × sex interactions at either locus (data not shown).

### Gene annotation and gene expression analysis

The genomic LD region surrounding the signal at 3p22.2 (approximately 415 kb, *r*^2^ > 0.3 with rs4453791, Figure 2c) contains a cluster of loci including *SCN11A*, *WDR48*, *GORASP1*, *TTC21A*, *CCSRN1* (also known as *AXUD1*), *XIRP1* and *CX3CR1*. There was no evidence that rs4453791 was related to coding variation within these genes, but there was LD with nearby regulatory sites (Figure 2c and Additional file 1: Table S5), especially near *CCSRN1* (rs1274963, *r*^2^ = 0.48) and *XIRP1* (rs17729892, *r*^2^ = 0.49; rs4676609, *r*^2^ = 0.33). Consistent with this observation, the study of nearby genes using LCL in ALSPAC (Additional file 1: Table S6) provided evidence for rs4453791 related, *cis*-acting expression alterations in *WDR48* (*P* = 0.00062), *GORASP1* (*P* = 0.0031), *XIRP1* (*P* = 0.0039) and *CCSRN1* (*P* = 0.0057). At each of these loci, the rs4453791 risk allele (C) was associated with a decrease in RNA expression.

The genomic area at 20p12.3 harbouring variants in LD with rs3761168 (approximately 33 kb) was restricted to the *PLCB1* locus itself (*r*^2^ > 0.3, Figure 3c). None of these variants was related to protein-coding variation, expression related changes in LCL (Additional file 1: Table S6) or strong alterations of nearby non-coding functional sites, although some evidence pointed to DNA motif changes (HaploReg v2: http://www.broadinstitute.org/mammals/haploreg/haploreg.php; data not shown).

### Search for potential autism spectrum disorder susceptibility loci

We investigated all SNPs contributing to independent, population-specific, social-communication related GWAS signals (8- and 17-year time-points; *P* ≤ 1 × 10^−5^) for their association with ASD within the AGRE and ACC samples in a search for underlying ASD quantitative trait loci (QTLs) affecting the entire spectrum. None of the signals revealed any association with ASD in both autism samples together (Additional file 1: Table S7).

The LD-based genomic region near the two strongest population-based GWAS signals at 3p22.2 and 20p12.3 (17-year time-point) harboured ASD susceptibility loci. rs4453791 was in LD with variants within *XIRP1* (a locus with weak ASD candidacy) [44], and rs3761168 was in LD with variants within *PLCB1*[45,46]. Permutation analysis (Table 2) performed to account for multiple testing and conditional on the set of known autism candidate genes suggested that the observed co-location of these common signals within the vicinity of ASD susceptibility loci is unlikely to be due to chance (empirical *P* ≤ 0.007 (SE = 0.0026)). Because of the small LD region at 20p12.3, containing only a single locus, both the probability of genome-wide association and autism candidacy could be assessed for the same gene, thus clearly pointing to *PLCB1* as a potential autism susceptibility locus. At 3p22.2, however, the situation is more complex, as the GWAS signal, due to LD, may refer to multiple loci, including *SCN11A*, and thus no single gene could be prioritised.

## Discussion

In this study, we conducted a genome-wide analysis of impaired social-communication traits at multiple time-points during childhood and adolescence within a large sample of children of European origin. Prior to evaluating single SNP associations using a GWAS approach, we conducted an analysis of overall measurable common genetic influences and environmental-residual factors affecting social-communication difficulties during development. By focussing our GWAS on the phenotypes with the highest GCTA heritability, we found evidence for common association signals, including variation within the vicinity of putative ASD loci carrying rare mutations.

GCTA yielded strong evidence for the contribution of measurable common genetic effects during childhood and especially during later adolescence, accounting for approximately one-fourth and almost one-half of the phenotypic variance, respectively. However, there was little to no evidence for such effects during early to middle adolescence. The observed drop in GCTA heritability is consistent with recent reports of low to zero GCTA heritability for childhood behaviour problems, including autistic symptoms, at the beginning of adolescence [47]. Overall, the observed GCTA estimates (*h*^2^ = 0.08 to 0.45) were considerably smaller than those in previous twin studies (*h*^2^ = 0.36 to 0.87 [7]). GCTA, however, captures additive genetic influences only and depends on the assumption that causal variation is sufficiently represented through the set of genotyped SNPs on the chip [33]; thus it may lack many rare variants [48]. Therefore, on average, GCTA heritability estimates are only about one-half the size of twin study heritability estimates [49].

GCTA-based genetic correlations, which capture the correlation between genetic effects independent of heritability and are relatively unbiased [50], indicated, however, some genetic stability between time points. Specifically, they suggested that about one-half of the genes affecting social-communication problems in early childhood and later adolescence remain the same (approximately 51% between ages 8 and 17 years), implying that some genetic effects may change over the long term. However, for adjacent time-points, the similarity between genetic influences was much higher (82% to 97%). Thus, we observed genetic stability combined with considerable variation in GCTA heritability for social-communication traits close in time. These apparently contradictory findings deserve a more detailed explanation. Heritability estimates are relative measures (that is, proportions) and not absolute measures of genetic influences; thus both genetic and environmental-residual variance components need to be taken into account. Given the strong genetic correlation between time points close in time, it is unlikely that variation in GCTA heritability is due to a sudden major change in the underlying genetic architecture. Rather, environmental-residual age-specific effects, including measurement error, may play a role in changing the underlying variance composition over time. Firstly, environmental-residual correlations were considerably lower than their genetic counterparts; secondly, both types of correlation decreased with progressing age. It can be speculated that such age-specific influences may be related to pubertal adjustments, including, for example, transitional behavioural and social problems during early to middle adolescence [51], adding ‘noise’ to variation in social-communication traits. Moreover, adolescent-parent related pubertal stress within families [52] may affect mothers’ reports regarding their children’s behaviour and skills. For example, using twin analysis, heritability of a related phenotype, language skills, declined from mid-childhood to mid-adolescence when indirectly assessed on the basis of teacher report [26], whereas it plateaued when language skills were directly measured. GCTA, however, cannot disentangle environmental from residual influences [33], nor can it characterise non-shared variation, thus highlighting the importance of twin study designs.

GCTA heritability estimates, however, can provide information on the power of GWAS to detect genetic influences, and GCTA-based correlations can capture the expected genetic heterogeneity between time points. This is because GWAS and GCTA are restricted by the same underlying limitations [50], including the measurement of additive genetic effects and the adequate representation of causal variation on genotyping platforms. In this study, therefore, we focussed our genome-wide association screen on the phenotypes with the highest GCTA heritability (ages 8 and 17 years). We conducted single time-point analyses, thus allowing for some genetic heterogeneity across time, and corrected for measurement relatedness and type I error through genome-wide permutations.

There was evidence for genome-wide association at 3p22.2 and near genome-wide association at 20p12.3 for the 17-year time point only. *Post hoc* longitudinal modelling showed that the signal at 3p22.2 was time-sensitive and marked by a continuous increase in genetic effect during development, which is consistent with some decline in genetic correlations over time. The association signal at 20p12.3 was developmentally more heterogeneous and might have been affected by noise.

The closest locus harbouring SNPs in LD with variation at 3p22.2 (rs4453791) was *SCN11A* [OMIM:604385]. The gene encodes the α subunit of voltage-gated sodium channels, which are membrane-protein complexes with an important role in the voltage-dependent sodium ion permeability of excitable membranes. Specifically, SCN11A mediates rapid brain-derived neurotropic factor-evoked membrane depolarisation via the receptor tyrosine kinase NTRK2 [53]. Intriguingly, rare mutations within genes encoding similar α subunits, such as *SCN1A*, *SCN2A* and *SCN3A*, have been linked to autism [54]. However, rs4453791 is also related to non-coding functional variation near *CCSRN1* and *XIRP1*, the latter being an autism susceptibility locus of weak candidacy [44].

The signal at 20p12.3 (rs3761168) was found at *PLCB1* [OMIM:607120] and was restricted by LD to this locus only. There was little evidence for a functional role of the associated SNP, although involvement in DNA motif changes is possible. PLCB1 is involved in extracellular signal transduction and catalyses the formation of inositol 1,4,5-trisphosphate and diacylglycerol from phosphatidylinositol 4,5-bisphosphate. The *PLCB1* locus has previously been established as a susceptibility locus for ASD, harbouring multiple rare mutations. This includes an ASD-specific, approximately 480 kb inherited deletion [45]. In addition, multiple deletions and duplications of ≥480 kb were found to be enriched in individuals with ASD compared to controls [46], and other rare deletions within *PLCB1* have been linked to early-onset epileptic encephalopathy [55] and schizophrenia [56].

Our search for dimensional ASD QTL found little evidence for the contribution of social-communication related signals to risk for autism, which is consistent with recent views on the role of common genetic variation in ASD [18]. However, our empirical analysis suggested that social-communication related associations are unlikely to be found within the vicinity of ASD susceptibility loci by chance. This supports the hypothesis that in particular *PLCB1*, a locus where LD is clearly defined, may play a more general functional role in the autism continuum with rare, protein-disrupting *PLCB1* mutations contributing to ASD and common *PLCB1* variation contributing to subtle changes in social-communication difficulties. Similar relationships, for example, have been demonstrated for low-density lipoprotein receptor (*LDLR*) gene mutations that cause familial hypercholesterolemia [22], as well as for common *LDLR* variants which are related to smaller elevations in cholesterol [23]. The reported findings thus support connections between a ‘rare allele model’ of complex diseases and a ‘common variant model’ of population-based traits.

The major strength of our study is the exploration of the common genetic architecture of social-communication traits over time, which can be assessed by current chip arrays and imputation efforts, and the exploitation of GCTA to enhance the power of subsequent genome-wide association screens. The identification of specific genetically enriched time-points, however, also limits the pool of available replication samples, as, for example, our main signal at 3p22.2 does not show any association at 11 years, an age at which data on social-communication phenotypes is available within many cohorts. Our study thus emphasises the need to collect social-communication phenotypes across development, including late adolescence and possibly adulthood. Given that genetically backed social-communication measures in near-adult populations are rare, we selected genome-wide permutations to control for type I error. This allowed us to adjust for measurement relatedness without modelling the genetically less enriched phenotypes, which might be affected by increased ‘measurement noise’. Although permutation analysis cannot provide the same robustness as replication in independent samples (and we therefore cannot entirely rule out type I error), at least one identified common signal within our study overlaps with a locus, which has been implicated within ASD through a rare disease mechanism, *PLCB1*.

## Conclusion

Together, our findings suggest that social-communication difficulties are developmentally characterised by variation in GCTA heritability, despite some genetic stability, and that these changes may affect detectable overlaps with the autism spectrum. An informed analysis of phenotypes with high GCTA heritability, however, may increase the power of genome-wide association screens.

### Supporting data

Supplementary information is provided as Additional file 1.

## Abbreviations

ASD: Autism spectrum disorder; ADI–R: Autism Diagnostic Interview–Revised; ACC: Autism Case Control cohort; AGRE: Autism Genetic Resource Exchange; ALSPAC: Avon Longitudinal Study of Parents and Children; GC: Genomic control; GCTA: Genome-wide Complex Trait Analysis; GWAS: Genome-wide association study; HapMapCEU: Utah residents with Northern and Western European ancestry from the Centre d’Etude du Polymorphisme Humain collection; LCL: Lymphoblastoid cell line; LD: Linkage disequilibrium; LDLR: Low-density lipoprotein receptor; MAF: Minor allele frequency; MDS: Multidimensional scaling; QTL: Quantitative trait locus; SCDC: Social Communication Disorder Checklist; SNP: Single-nucleotide polymorphism.

## Competing interests

The authors declare that they have no competing interests.

## Authors’ contributions

BSP and KW carried out the statistical analysis. BSP, DME, JPK, SMR and WLM were involved in the preparation of the genotype information. BSP, DHS, WPM and GDS participated in the design of the study. BSP, DHS, WPM, KW, HH, NJT, DMW, JPK, JG and GDS helped to draft the manuscript. All authors read and approved the final manuscript.

## Supplementary Material

Additional file 1 **Additional Note.** Genome-wide Complex Trait Analysis. **Table S1.** Temporal stability of social-communication problems. **Table S2.** Genetic correlations. **Table S3.** Genome-wide association signals for social-communication problems at single time-points. **Table S4.** Longitudinal analysis of the strongest single time-point association signals. **Table S5.** Functional characterisation of non-coding variation near rs4453791. **Table S6.** Expression quantitative trait locus analysis. **Table S7.** Follow-up analysis of social-communication related signals in autism samples. **Figure S1.** Quantile-quantile plots of genome-wide association signals. Click here for file
