# Supplementary material for: Variability in the common genetic architecture of social-communication spectrum phenotypes during childhood and adolescence
Source: Mol Autism. 2014 Feb 24;5:18. doi: 10.1186/2040-2392-5-18 (PMC3940728; doi:10.1186/2040-2392-5-18)
Supplement: Additional file 1 — Additional Note. Genome-wide Complex Trait Analysis. Table S1. Temporal stability of social-communication problems. Table S2. Genetic correlations. Table S3. Genome-wide association signals for social-communication problems at single time-points. Table S4. Longitudinal analysis of the strongest single time-point association signals. Table S5. Functional characterisation of non-coding variation near rs4453791. Table S6. Expression quantitative trait locus analysis. Table S7. Follow-up analysis of social-communication related signals in autism samples. Figure S1. Quantile-quantile plots of genome-wide association signals. [file 2040-2392-5-18-S1.docx]

**Variability in the common genetic architecture of social-communication spectrum phenotypes during childhood and adolescence**

Beate St Pourcain PhD, David H. Skuse MD, William P. Mandy PhD, Kai Wang PhD, Hakon Hakonarson MD PhD, Nicholas J. Timpson PhD, David M. Evans PhD, John P. Kemp PhD, Susan M. Ring PhD, Wendy L. McArdle PhD, Jean Golding PhD DSc, George Davey Smith MD DSc

**Additional Material**

1. **Additional Note**

Genome-wide Complex Trait Analysis

1. **Additional Tables**

Table S1: Temporal stability of social-communication problems

Table S2: Genetic correlations

Table S3: Genome-wide association signals for social-communication problems at single time-points

Table S4: Longitudinal analysis of the strongest single time-point association signals

Table S5: Functional characterisation of non-coding variation near rs4453791

Table S6: Expression quantitative trait locus analysis

Table S7: Follow-up analysis of social-communication related signals in autism samples

1. **Additional Figure**

Figure S1: Quantile-quantile plots of genome-wide association signals

**Additional Notes**

**Genome-wide Complex Trait Analysis**

An estimation of the proportion of additive phenotypic variation explained by all SNPs together (narrow-sense GCTA heritability) was performed for social-communication problems at 8, 11, 14 and 17 years of age using ‘Genome-wide Complex Trait Analysis’ (GCTA)[1]. Based on a sample of independent individuals, this method captures the trait variance, which is tagged when all SNPs are considered simultaneously. This is achieved by comparing a matrix of pairwise genomic similarity with a matrix of pairwise phenotypic similarity using a random-effects mixed linear model[1]. Pertinent to this study, GCTA was performed using rank-transformed (and thus normally distributed) residuals of social-communication traits adjusted for age, sex and the first two principal components, and 464,311 directly genotyped SNPs. The extent to which the same genes or environmental-residual factors contribute to the observed phenotypic correlation between two variables can be estimated through genetic and environmental-residual correlation respectively[2]. Bivariate GCTA [3] was carried out to estimate the genetic correlation (r_g_) between each measured time-point (based on the genetic covariance between two traits) and their environmental-residual correlation (r_e_, based on the residual covariance). Note that GCTA does *not* distinguish between environmental and residual variation. The environmental-residual correlation can be estimated as r_e_=C_e_/(√V_e1_* √ V_e2_), where C_e_ is the residual covariance between traits 1 and 2, and V_e1_ and V_e2_ are the residual variances of trait 1 and 2 respectively. As the GCTA software does not provide the standard error for r_e_, it was estimated as Var(r_e_) = r_e_*r_e_*(VarV_e1_/(4*V_e1_*V_e1_)+VarV_e2_/(4*V_e2_*V_e2_)+VarC_e_/(C_e_*C_e_) +CovV_e1_V_e2_/(2*V_e1_*V_e2_)-CovV_e1_C_e_/(V_e1_*C_e_)-CovV_e2_C_e_/(V_e2_*C_e_)) and SE(r_e_) = √Var(r_e_), where VarV_e1_ and VarV_e2_ are the sampling variances for V_e1_ and V_e2_ respectively, VarC_e_ is the sampling variance for C_e_, CovV_e1_V_e2_ is the sampling covariance between V_e1_ and V_e2_, CovV_e1_C_e_ is the sampling covariance between V_e1_ and C_e_, and CovV_e2_C_e_ is the sampling covariance between V_e2_ and C_e_ [4, 5] (Liang Yang, personal communication). The relationship between the phenotypic correlation (r_p_) in two traits 1 and 2, their trait heritabilities (h^2^) and their environmentalities (e^2^, proportion of phenotypic variance that is attributable to environmental-residual variance), the genetic correlation r_g_, and the environmental correlation r_e_, assuming no gene-environment interactions or correlations, can be described as r_p_=h_1_*h_2_*r_g_ + e_1_*e_2_*r_e_, where h_1_ and h_2_ correspond to the square root of the heritabilities, and e_1_ and e_2_ correspond to the square root of the environmentalities[6].

**References**

1. Yang J, Manolio TA, Pasquale LR, Boerwinkle E, Caporaso N, Cunningham JM, de Andrade M, Feenstra B, Feingold E, Hayes MG, Hill WG, Landi MT, Alonso A, Lettre G, Lin P, Ling H, Lowe W, Mathias RA, Melbye M, Pugh E, Cornelis MC, Weir BS, Goddard ME, Visscher PM: **Genome partitioning of genetic variation for complex traits using common SNPs**. *Nat Genet* 2011, **43**:519–525.

2. Neale MC, Maes HHM: *Methodology for Genetic Studies of Twins and Families.* Dordrecht, The Netherlands: Kluwer Academic Publishers B.V.; 2004.

3. Lee SH, Yang J, Goddard ME, Visscher PM, Wray NR: **Estimation of pleiotropy between complex diseases using single-nucleotide polymorphism-derived genomic relationships and restricted maximum likelihood**. *Bioinformatics* 2012, **28**:2540–2542.

4. Lynch M, Walsh B: *Genetics and Analysis of Quantitative Traits.* Sinauer Associates Inc.,U.S.; 1998.

5. Trzaskowski M, Yang J, Visscher PM, Plomin R: **DNA evidence for strong genetic stability and increasing heritability of intelligence from age 7 to 12**. *Mol Psychiatry* 2013. 10.1038/mp.2012.191

6. Fuller JL, Thompson WR: *Foundations of Behaviour Genetics.* St Louis, MO: Mosby; 1978.

**Additional tables**

**Table S1:** Temporal stability of social-communication problems

| **Age in years** | | | | |
| --- | --- | --- | --- | --- |
|  | **8** | **11** | **14** | **17** |
| **8** | 1.00 | 0.61 | 0.50 | 0.38 |
| **11** | 0.57 | 1.00 | 0.57 | 0.41 |
| **14** | 0.49 | 0.57 | 1.00 | 0.51 |
| **17** | 0.39 | 0.45 | 0.56 | 1.00 |

Lower triangle: Spearman’s rank correlation using pairwise complete observations

Upper triangle: Pearson product-moment correlation using rank-transformed measures of social-communication problems adjusted for age, sex and the two most significant principal components

**Table S2:** Genetic correlations

| **Age in years** | | | | |
| --- | --- | --- | --- | --- |
|  | **8** | **11** | **14** | **17** |
| **8** | - | 7x10^-5^ | 0.04 | 0.0008 |
| **11** | 0.97(0.14) | - | 0.03 | 0.01 |
| **14** | 0.68(0.32) | 0.82(0.27) | - | 2x10^-7^ |
| **17** | 0.51(0.14) | 0.40(0.16) | 0.95(0.36) | - |

Analyses were performed on rank-transformed measures of social-communication problems adjusted for age, sex and the most significant principal components, individuals with a relatedness of ≥2.5% were excluded, GCTA – Genome-wide Complex Trait Analysis

Lower triangle: Genetic correlations and their standard errors (SE) were estimated using bivariate GCTA

Upper triangle: Associated P-value (GCTA-based likelihood ratio test with H0: r_g_=0)

**Table S3:** Genome-wide association signals for social-communication problems at single time-points

| **Age (years)** | **SNP** | **Chr** | **Gene** | **Autism locus^a^** | **E,A** | **EAF** | **I/G** | **β(SE)** **^b^** | **P^b^** |
| --- | --- | --- | --- | --- | --- | --- | --- | --- | --- |
| 8 | rs1581057 | 3 | intergenic | - | c,a | 0.69 | I | 0.13(0.03) | 5.8x10^-6^ |
| 8 | rs9942541 | 6 | *KCNK5* | - | t,c | 0.05 | I | 0.23(0.05) | 5.1x10^-6^ |
| 8 | rs4460308 | 7 | *LHFPL3* | - | c,t | 0.36 | G | 0.11(0.03) | 5.8x10^-6^ |
| 8 | rs2839874 | 9 | *COL27A1* | - | c,g | 0.73 | I | 0.13(0.03) | 6.1x10^-6^ |
| 8 | rs12342373 | 9 | *LMX1B* | *LMX1B* | a,g | 0.09 | I | 0.19(0.04) | 2.8x10^-6^ |
| 8 | rs1557765 | 11 | *KCNJ11* | - | c,t | 0.63 | G | 0.12(0.03) | 7.0x10^-6^ |
| 8 | rs11109142 | 12 | *AF429306* | - | g,c | 0.03 | I | 0.31(0.07) | 4.4x10^-6^ |
| 8 | rs4905226 | 14 | *SERPINA13* | - | t,c | 0.24 | G | 0.13(0.03) | 3.7x10^-6^ |
| 8 | rs17828380 | 15 | *RAB8B* | - | c,g | 0.11 | I | 0.18(0.04) | 5.4x10^-6^ |
| 8 | rs7199390 | 16 | *C16orf75* | - | t,a | 0.10 | I | 0.19(0.04) | 2.3x10^-6^ |
| 8 | rs17750321 | 18 | *BRUNOL4* | - | a,c | 0.03 | I | 0.3(0.06) | 5.4x10^-6^ |
| 17 | rs2304003 | 2 | *KIAA1992* |  | t,c | 0.26 | I | 0.14(0.03) | 8.6x10^-6^ |
| **17** | **rs4453791** | **3** | ***SCN11A*** | ***XIRP1*** | **c,t** | **0.13** | **I** | **0.23(0.04)** | **9.3x10^-9^** |
| 17 | rs11819364 | 10 | *DOCK1* |  | c,a | 0.03 | G | 0.32(0.07) | 8.7x10^-6^ |
| 17 | rs4622507 | 16 | *IRX5* |  | c,t | 0.26 | G | 0.15(0.03) | 2.4x10^-6^ |
| 17 | rs1539809 | 18 | *EPB41L3* |  | t,c | 0.04 | I | 0.33(0.07) | 1.7x10^-6^ |
| 17 | rs3761168 | 20 | *PLCB1* | *PLCB1* | a,c | 0.05 | I | 0.32(0.06) | 7.9x10^-8^ |

Results are presented for independent loci with GC corrected *P* ≤ 10^-5^ (LD-based clumping: r^2^>0.3, ±500 kb). Regression estimates were obtained using quasi-Poisson regression. Gene – Nearest gene within ±500 kb of the SNP; E – Effect allele, A – Alternative allele, EAF – Effect allele frequency; I/G – Imputed/Genotyped; LD - Linkage disequilibrium; All SNPs had an imputation quality of 0.80 <R^2^<0.99 (MaCH); Genome-wide significant results are indicated in bold

a – Autism candidate locus in LD (<http://sfari.org/>)

b - Genomic-control (GC) corrected

**Table S4**: Longitudinal analysis of the strongest single time-point association signals

| **SNP** | **Fixed effects** | **β(SE)** | **Z** | **P** |
| --- | --- | --- | --- | --- |
| rs4453791_C | rs4453791 x age | 0.02(0.005) | 3.21 | 0.0013 |
|  | rs4453791 at age 8 years^a^ | 0.032(0.039) | 0.83 | 0.41 |
|  | rs4453791 at age 11 years^a^ | 0.085(0.035) | 2.43 | 0.015 |
|  | rs4453791 at age 14 years^a^ | 0.14(0.038) | 3.63 | 0.00028 |
|  | rs4453791 at age 17 years^a^ | 0.19(0.047) | 4.06 | 4.9x10^-5^ |
| rs3761168_A | rs3761168 | 0.17(0.053) | 3.3 | 9.8x10^-4^ |

a – Fixed SNP effects at different age ranges

Longitudinal analysis was based on a multilevel Poisson model. There was no support for SNP × sex interactions at either locus (data not shown).

**Table S5:** Functional annotation of non-coding variation near rs4453791

| **SNP** | **r^2^** | **Gene** | **Regulome** | **eQTL** | **TF motif** | **Histone modification (ChIP seq)** | **Protein binding (ChIP seq)** | **DNase seq** |
| --- | --- | --- | --- | --- | --- | --- | --- | --- |
| rs1274963 | 0.48 | *CCSRN1* | 1d | RPSA (lymphoblastoid) | EWSR1-FLI1 | Yes(Multiple) | POLR2A(K562) | Yes(K562) |
| rs4676609 | 0.33 | *XIRP1* | 1f | RPSA (lymphoblastoid) | - | Yes(Multiple) | - | Yes(T47d) |
| rs17729892 | 0.49 | *XIRP1* | 2b | - | AIRE | Yes (HSMM ) | EGR1(K562) | Yes(Multiple) |

r^2^ – Linkage disequilibrium (r^2^) coefficient with respect to rs4453791

*Annotation is only given for variants with strong evidence for functional non-coding variation* (i.e. Regulome codes 1 or 2: 1 - Likely to affect binding of a protein to DNA and linked to expression of a gene target, 2 - Likely to affect binding of a protein to DNA; <http://regulome.stanford.edu/>); Regulome – Regulome database score: 1d - eQTL + TF binding + any motif + DNase peak;1f - eQTL + TF binding / DNase peak ; 2b - TF binding + any motif + DNase footprint + DNase peak; eQTL - Expression quantitative trait locus related to SNP variation; TF – Transcription factor binding motif; ChIP seq - Chromatin immunoprecipitation (ChIP) with massively parallel DNA sequencing to identify the binding sites of DNA-associated proteins and histone modifications; DNase seq – DNase I hypersensitivity as identified by DNase I hypersensitive sites sequencing; Information on cell lines are given in parentheses (H1 - Embryonic stem cells; HSMM - Skeletal muscle myoblasts; K562 – Leukaemia cell line; T47D - Human ductal breast epithelial tumor cell line)

**Table S6:** Expression quantitative trait locus analysis

| **SNP** | **Transcript**^a,^**^b^** | **Illumina probe^c^** | **β(SE)** | **P** |
| --- | --- | --- | --- | --- |
| rs4453791_C | *SCN11A* | ILMN_1797892 | 0.04(0.07) | 0.52 |
|  | *WDR48* | ILMN_1762103 | -0.24(0.07) | 0.00062 |
|  | *TTC21A* | ILMN_1715332 | -0.14(0.07) | 0.052 |
|  | *GORASP1* | ILMN_1716821 | -0.21(0.07) | 0.0031 |
|  | *CCSRN1* | ILMN_1703123 | -0.19(0.07) | 0.0058 |
|  | *XIRP1* | ILMN_1802160 | -0.2(0.07) | 0.0039 |
|  | *CX3CR1* | ILMN_2088437 | -0.07(0.07) | 0.28 |
|  |  | ILMN_1745788 | 0.03(0.07) | 0.65 |
| rs3761168_A | *PLCB1* | ILMN_1723969 | -0.16(0.1) | 0.12 |
|  |  | ILMN_1708432 | -0.03(0.1) | 0.79 |

Expression quantitative trait locus (eQTL) analysis of *cis* transcript expression in lymphoblastoid cell lines

a – e-QTL analysis based on up to 875 unrelated ALSPAC individuals

b – Gene within LD-based gene region (r^2^>0.3 HapmapCEU(release 22))

c – Illumina HT-12 v3 bead array

**Table S7:** Follow-up analysis of social-communication related signals in autism samples

|  |  |  | **AGRE** | | | **ACC** | | |
| --- | --- | --- | --- | --- | --- | --- | --- | --- |
| SNP | Chr | E,A | EAF | Z | P | EAF^a^ | OR(95% CI) | P |
| rs1581057 | 3 | c,a | 0.67 | -0.75 | 0.45 | 0.69 | 1.03(0.94,1.13) | 0.42 |
| rs9942541 | 6 | t,c | 0.06 | 0.24 | 0.81 | 0.05 | 0.96(0.78,1.18) | 0.86 |
| rs4460308 | 7 | c,t | 0.35 | -0.82 | 0.41 | 0.35 | 0.98(1.07,0.89) | 0.65 |
| rs2839874 | 9 | c,g | 0.73 | 0.71 | 0.48 | 0.75 | 1.12(1.25,1.01) | 0.036 |
| rs12342373 | 9 | a,g | 0.09 | -1.57 | 0.12 | 0.09 | 1.07(1.24,0.92) | 0.51 |
| rs1557765 | 11 | c,t | 0.60 | -0.78 | 0.44 | 0.62 | 0.95(1.04,0.87) | 0.24 |
| rs11109142^a^ | 12 | g,c | 0.02 | 0.11 | 0.91 | 0.01 | 0.69(0.48,0.99) | 0.24 |
| rs4905226 | 14 | t,c | 0.24 | -0.55 | 0.58 | 0.23 | 0.91(0.82,1.00) | 0.060 |
| rs17828380 | 15 | c,g | 0.10 | -0.07 | 0.94 | 0.09 | 0.99(1.15,0.85) | 0.96 |
| rs7199390 | 16 | t,a | 0.08 | 1.19 | 0.23 | 0.08 | 1.05(0.89,1.23) | 0.33 |
| rs17750321 | 18 | a,c | 0.02 | -0.85 | 0.40 | 0.02 | 1.02(1.41,0.74) | 0.86 |
| rs2304003 | 2 | t,c | 0.25 | -0.45 | 0.65 | 0.25 | 0.99(0.90,1.09) | 0.93 |
| rs4453791 | 3 | c,t | 0.13 | 0.71 | 0.48 | 0.12 | 1.01(1.17,0.88) | 0.66 |
| rs11819364 | 10 | c,a | 0.03 | 0.31 | 0.76 | 0.03 | 1.03(0.81,1.31) | 0.81 |
| rs4622507 | 16 | c,t | 0.29 | 0.73 | 0.47 | 0.29 | 1.05(1.16,0.95) | 0.35 |
| rs1539809 | 18 | t,c | 0.02 | 1.70 | 0.090 | 0.02 | 0.98(0.73,1.31) | 0.49 |
| rs3761168 | 20 | a,c | 0.07 | -0.24 | 0.81 | 0.05 | 0.89(1.08,0.73) | 0.13 |

Family-based association analysis was performed with FBAT using the most likely genotypes; Case-Control association analysis was conducted using SNPTEST; All SNPs had sufficient imputation quality (AGRE: 0.73 <R^2^≤1 (MaCH) ; ACC: 0.75 <PROPER_INFO≤1 (SNPTEST)); AGRE – Autism genetic research exchange (AGRE) sample (793 ASD pedigrees); ACC – Autism Case-Control cohort (1204 ASD subjects, 6491 control subjects); E – Effect allele, A – Alternative allele, EAF – Effect allele frequency; 95%-CI – 95% Confidence interval

a – Within ASD subjects

**Additional Figure**

**Figure S1:** Quantile-quantile plots of genome-wide association signals

Genome-wide analysis of social-communication difficulties in ALSPAC at 8 years (a) (λ=1.04) and 17 years (b) (λ=1.03) of age. Black circles depict the observed association signals (Genomic-control corrected), the white diagonal line represents the distribution of signals under the null hypothesis and the shaded area corresponds to the 95% confidence interval. A deviation of the observed from the expected distribution of signals is visible for social-communication related signals at age 17 years.

λ – Genomic-control factor
